# The development of the Chinese version of the Sports Emotional Intelligence Scale

**DOI:** 10.3389/fpsyg.2022.984426

**Published:** 2022-10-03

**Authors:** Jia Zhang, Donghuan Bai, Long Qin, Pengwei Song

**Affiliations:** ^1^School of Physical Education, Chongqing University, Chongqing, China; ^2^School of Physical Education, Huaibei Normal University, Huaibei, China; ^3^School of Physical Education, Guangxi Science and Technology Normal University, Laibin, China; ^4^School of Physical Education, Keimyung University, Daegu, South Korea

**Keywords:** sports emotional intelligence, reliability, validity, scale revision, Chinese version

## Abstract

**Objective:**

To revise and test the Chinese version of the Sports Emotional Intelligence Scale (SEIS) in sports situations.

**Materials and methods:**

After pretesting 112 college students, 832 college students were formally tested, and item analysis, validity test, internal consistency reliability analysis, and calibration validity and equivalence test of the Chinese version of the SEIS were performed. The Chinese version of the SEIS had 14 items with four dimensions (evaluation of others’ emotions, self-emotional management, emotion use, and social skills), with a cumulative variance contribution of 57.812 percent; the four-factor measurement model fit well (χ^2^/df = 3.743, NFI = 0.917, GFI = 0.945, AFGI = 0.913, CFI = 0.926, and RMSEA = 0.062). The internal consistency coefficients (Cronbach’s α) of the Chinese version of the SEIS ranged from 0.824 to 0. 905, and the split-half reliability ranged from 0.807 to 0.891; the correlations between the total SEIS score and its dimensional scores and the calibration variables were significantly correlated (*p* < 0.05), ranging from 0.255 to 0.603. The gender measure equivalence test was valid, and the mean difference of potential variables across gender groups was not significant in the potential mean analysis.

**Conclusion:**

The Chinese version of the SEIS has good reliability and validity, and it is appropriate for assessing emotional intelligence in sports situations.

## Introduction

According to Salovey and Mayer (1990), who first put out the concept, emotional intelligence refers to a person’s capacity for logical thought and blends emotional and cognitive skills (including learning, memory, reasoning, and judgment) with emotional qualities (like happiness and sorrow) (Gersimova, 2018). Where the dynamic interplay of emotion-cognition-behavior exemplifies the fundamental idea of emotional intelligence (Vaida, 2014). That is, individuals think alongside their emotions, which modify the individual’s emotions, and the changing emotions act on the individual’s cognitive system, which determines the individual’s synchronous action (Ma et al., 2021). Emotional intelligence is defined as the perception of emotions, their appraisal and performance, their reasoning, their comprehension and analysis, and their regulation (Mitic et al., 2021). Previous research has shown that emotional intelligence enables people to effectively overcome frustrating and stressful situations and improves personal executive ability to correctly perceive and understand their emotions (Gupta and Bajaj, 2018), as well as having the ability to regulate their emotions based on the situation and use them constructively, such as motivating themselves and shifting their attitudes in a positive direction (Gonzalez et al., 2010). When there is a good knowledge of emotions and proper adjustment of negative emotions, strong emotional intelligence will make it easier to recover more quickly from negative emotions and express emotions more consistently (Gao, 2020). Emotional intelligence is seen as a vital internal resource that may assist individuals in regulating their emotions, managing interpersonal relationships, and improving their physical and mental health and well-being.

Over the years, related scholars have sought to uncover how sport participants’ emotional experiences impact the execution of sport behaviors, which has greatly contributed to the development of theoretical understanding on sport emotions. one of the meta-emotional theories that incorporate the cognitive processes, functions, and capabilities of emotions has been applied. Meta-emotion theory is an integrative emotion-cognition-behavior theory that proposes that emotions have adaptive functions or activity tendencies that arbitrate subsequent behavior and generate energy by interacting with cognition (Liang et al., 2020), where emotional intelligence can be seen as the central mechanism of meta-emotion theory and includes the ability to evaluate and express one’s own and others’ emotions, the ability to regulate emotions effectively, an ability to regulate emotions effectively (He et al., 2020).

In sport situations, incoming emotional information essentially undergoes a three-stage cognitive information processing process (awareness/understanding, management/regulation, and utilization/facilitation) to be outputted in the form of optimally motivated behaviors (Yoo, 2010; Zurita and Ramirez, 2020). This provides a framework for exploring the structure and function of the emotional intelligence of sport participants in sporting contexts. It has been shown that sport participants experience multiple emotions during physical activity and that sports emotional intelligence can determine their sports behavioral tendencies and physical activity execution based on the characteristics of the emotions experienced and their emotional regulation or control, and can be used as a prior variable to predict sports participants’ behavioral tendencies (Sung et al., 2010), sports performance (Zhao et al., 2016), or sports burnout (Rizvandi et al., 2020). High sports emotional intelligence participants will have a meta-emotional function that identifies and regulates the unique emotions that arise during physical activity and uses them positively for individual and team performance (Choi and Kim, 2014). In sporting contexts where people require high levels of motivation to continue to participate in physical activity, and where most (Table 1) forms of physical activity involve some degree of interpersonal interaction (e.g., other participants in the gym, coaches, etc.) (Acebes et al., 2021a), physical behavior is likely to be governed by emotional intelligence (Ortega et al., 2018). There is growing evidence that emotional intelligence plays an important role in exercise performance (Kopp et al., 2021) and physical activity, so constructing an understanding of emotional intelligence in sporting contexts is particularly important for the practice of empirical interventions that aim to improve exercise behavioral engagement or exercise adherence. Emotional intelligence is not an innate talent or trait, but is developed through learning (Lei et al., 2021), and some studies suggest that emotional education strategies to maximize emotional regulation (Vaquero et al., 2020) may be used as a way to enhance physical activity execution and self-determined levels of motivation.

**TABLE 1:** Descriptive statistics and correlations.

Title	Title—total score related	*t*
(1) I am very good at observing people’s emotions.	0.71	22.08
(2) I can have a very keen insight into the feelings and emotions of others.	0.65	18.25
(3) I am very aware of the emotions of the people around me.	0.74	26.77
(4) I can usually guess my friends’ emotions from their behavior.	0.70	21.45
(5) In a positive mood, I have so many new and exciting ideas.	0.66	18.74
(6) Problem solving comes easily to me in a positive mood.	0.67	19.62
(7) During physical exercise, I often know why I feel happy or unhappy.	0.73	25.26
(8) When faced with difficulties in physical activity, I will use positive thoughts or emotions to get through them.	0.73	24.92
(9) I can usually tell the reasons why I will feel certain ways.	0.68	20.90
(10) I know the reasons for the emotional changes I’ve experienced.	0.72	24.67
(11) I often know why I feel happy or unhappy.	0.75	27.42
(12) When exercising in sports, I usually prepare activities that will make my peers or coach happy.	0.71	22.71
(13) I prefer to share my emotions with my peers or coaches when exercising in sports.	0.66	19.11
(14) When my partner or coach is depressed, I help ease his emotions.	0.62	17.84

Foreign scholars have conducted in-depth research on the concept and model of emotional intelligence from different perspectives and formed two views represented by the ability model and the mixed model. The ability model views emotional intelligence as the ability to cognize and understand emotional information (Mayer and Salovey, 1997), while the mixed model view considers emotional intelligence as the ability to give oneself motivation, to endure in the face of frustration, to control impulsivity, to delay gratification, to regulate mood, to empathize with the emotions of others, and to have hope (Bar-On, 1997). Since the theoretical model of emotional intelligence was proposed, scholars from various countries have refined various measurement tools based on different models. Mayer and Salovey (1997) developed the MEZS (Multifactor Emotional Intelligence Scale) scale based on the theory of emotional intelligence ability model, which includes four dimensions: emotion perception, emotion activation, emotional understanding, and emotional regulation, with 402 items, but this scale not only takes considerable time to measure, but also does not present satisfactory empirical evidence for the emotional intelligence ability model. In the same year, Bar-On (1997) developed the Emotional Intelligence Scale (EQ-i, Emotional Quotient Inventory) consisting of 133 items on five dimensions (intrapersonal intelligence, personal intelligence, stress management, adaptability, and general emotion) based on the theory of the mixed model of emotional intelligence. Subsequently, Mayer et al. (2002) challenged the EQ-i Emotional Intelligence Inventory, arguing that the EQ-i Emotional Intelligence Inventory does not reflect the notion that emotional intelligence is an ability that is learned and developed over time and experience. Based on this, Mayer et al. developed the MSCEIT (Mayer-Salovey-Caruso Emotional Intelligence Test) to assess four emotional intelligence dimensions (emotion recognition, thought facilitation, emotion comprehension, and emotion management) (Mayer et al., 2002) and this scale is widely used.

Regarding the measurement of sport emotional intelligence in sporting contexts, the Emotional Intelligence Scale (EIS) developed by Schute et al. (1998) is more widely used, and this scale consists of 33 items on four dimensions (emotion perception, emotion use, self-emotion management, and emotion management of others) (Schute et al., 1998). However, Meyer and Fletcher (2007) strongly criticized sport emotional intelligence researchers for misusing inappropriate emotional intelligence concepts and measurement instruments and suggested that there is an urgent need to define, conceptualize, and standardize the measurement of sports emotional intelligence (Meyer and Fletcher, 2007). The Sport Emotional Intelligence Scale (SEIS) was later developed through repeated tests and revisions of the Sports Emotional Intelligence Scale by Lane et al. (2009), Yoo (2010), and Nam (2015), and others. This scale uses four dimensions, such as others’ emotion evaluation, self-emotion management, emotion regulation, and social skills, as the basic concepts of emotional intelligence in sport situations, and consists of 14 items, which are more often applied in the research of measuring emotional intelligence in sport situations.

In recent years, relevant scholars have devoted themselves to the exploration of the direction of emotional intelligence enhancement, and emotional intelligence education and emotional intelligence enhancement program development have begun to attract attention. Pioneering research has shown that emotional intelligence is a key factor in the successful life of an individual (Stough et al., 2009), and the need for emotional intelligence research has also been emphasized in physical education contexts (Meyer and Fletcher, 2007; Castro et al., 2018). Despite the recognition of the value and role of emotional intelligence in physical activity, the role of emotional intelligence in role recognition in empirical physical activity is limited. Interest in sport emotional intelligence research is increasing abroad in the field of sport psychology, but most domestic research related to sports emotional intelligence uses models and scales of emotional intelligence developed in organizational or educational contexts. Therefore, this study will revise a set of sports emotional intelligence scales that are applicable to the measurement of sports emotional intelligence suitable for the socio-cultural context in China based on the basic view of emotional intelligence ability models, with reference to the Sports Emotional Intelligence Scale (SEIS), and conduct reliability tests to provide reliable research on emotional intelligence in sport contexts. The study is intended to provide a reliable measurement tool for research on emotional intelligence in sports contexts.

## Materials and methods

### Study design

This study sample was drawn from two universities in Henan Province, and the questionnaires were distributed in classes and administered in groups, and were collected on the spot after completion of the questionnaires. The sample consisted of three parts: sample 1 was a pretest sample, 120 questionnaires were distributed, and 112 valid questionnaires were obtained by deleting all kinds of invalid questionnaires such as empty questions, multiple answers for the same item, and regular answers. There were 36 freshmen, 51 sophomores, and 25 juniors; 63 males and 49 females; the subjects’ ages ranged from 17 to 22 years old (Mage = 19.35 years, SD = 1.03); Sample 2 was the official administration sample, 955 questionnaires were distributed and 832 were valid. There were 259 freshmen, 264 sophomores, 221 juniors, and 88 seniors; 475 males and 357 females; the subjects’ ages ranged from 17 to 24 years old (Mage = 19.62 years, SD = 1.25); Sample 3 was a retest sample of 83 individuals randomly selected from sample 2 after a 4-week interval, 46 males and 37 females, all sophomores.

The study was approved by the Research Ethics Committee of the first author’s institution. Informed consent was obtained from the participants and school administrator before the data collection. Participants were informed that their participation was completely voluntary and they could terminate it at any time.

### Sports Emotional Intelligence Scale

The Sports Emotional Intelligence Scale (SEIS), revised by Nam (2015), was used to measure emotional intelligence in sports situations with sports participants. The scale contains 4 dimensions including other people’s emotion evaluation, self-emotion management, emotion use, and social skills and consists of 14 items. A 7-point Likert scale was used, with higher scores indicating higher levels of sports emotional intelligence.

In this study, this scale was translated, and the scale Chinese-ization process used the return-to-translate method (Mao et al., 2021). One English major researcher translated the original version of the scale into Chinese, and another high-level English major researcher translated it into English, and then asked English major teachers to compare the English and Chinese versions so that the content expression did not contradict the original English meaning but was clear and easy to understand. The phrases in the scale that do not conform to Chinese expression habits were adjusted. For example, item1: “I can tell someone’s emotion just by looking at their face.”, the expression is too absolutely, so in order to avoid misunderstanding, it was modified to “I am very good at observing people’s emotions.” During the process, the translator focused on analyzing the differences and disagreements in the language, and made minor modifications and adjustments to the Chinese version of the scale, and finally formed the initial version of the Chinese version of the SEIS. Afterward, 120 students were selected to fill out the initial version of the questionnaire, and the analysis revealed that the internal consistency of the four dimensions of others’ emotion evaluation, self-emotion management, emotion use, and social skills were 0.849, 0.905, 0.858, and 0.824 in order, and the internal consistency of the total scale was 0.873, and the reliability index of the initial version of the questionnaire met the requirements of psychometrics (Kline, 2011).

### Criterion-related validity scale

The Emotional Intelligence has been shown to have strong Criterion-related validity (Livingstone and Day, 2005). To measure Criterion-related validity, researchers typically examine the relationship of the target measure against other well-established, reliable, and valid measures of the same construct (convergent validity) and sometimes also how different the target measure is from measures of a different construct (divergent validity) (McMorris and Perry, 2015). Related studies have demonstrated the relevance of sports emotional intelligence to exercise behavior (Ubago et al., 2019). Given that the Exercise input scale, Exercise Adherence Scale, and Exercise Commitment Scale are widely accepted and have high reliability in China, they were chosen as validity scales for this study.

#### Exercise input scale

Reference was made to the Exercise Engagement Scale for College Students developed by Dong (2017), which consists of 20 items in four dimensions, including vigorous persistence, focused satisfaction, perception of values, and autonomy (Dong, 2017). The scale is scored on a 5-point Likert scale, with higher scores indicating higher levels of exercise engagement. The internal consistency coefficient of this questionnaire in this study was 0.878.

#### Exercise adherence scale

The outdoor exercise adherence scale for adolescents developed by Liu et al. (2011) was used, and the word “outdoor exercise” in the title was modified to “physical activity” according to the meaning of the question. The scale consists of 6 items and is scored on a 5-point Likert scale, with higher scores indicating higher levels of exercise adherence. The internal consistency coefficient of this questionnaire in this study was 0.835.

#### Exercise commitment scale

Referring to the revised Exercise Commitment Scale by Chen and Li (2006), the scale consists of 15 items in 5 dimensions, including exercise commitment, exercise enjoyment, personal commitment, social constraint, and opportunity to participate. The scale is scored on a 5-point Likert scale, with higher scores indicating higher levels of exercise commitment. The internal consistency coefficient of this questionnaire in this study was 0.851.

### Statistical analysis

Item analysis, reliability analysis and exploratory factor analysis were performed using IBM SPSS Statistics for Windows version 22 (IBM Corp., Armonk, NY, USA) for the Chinese version of SEIS, and validation factor analysis, equivalence testing and latent mean analysis were performed using AMOS 24.0 software. The accepted level of significance was *p* < 0.05.

## Results

### Item analysis

Item analysis was conducted in terms of the relationship between each topic and the total scale score. The subjects in Sample 2 were ranked in descending order by total scale score, and the 27% of the data ranked before and after were divided into high and low subgroups for independent samples *t*-tests. The results showed that each topic resulted in significantly higher scores for the high subgroup than for the low subgroup (*p* < 0.001). Also, the Pearson product difference correlation coefficient for each topic score and the mean score of the dimension to which it belongs ranged from 0.62 to 0.75, all at a statistically significant level (*p* < 0.001). Thus, all items were well discriminated and all 14 items were retained (Table 1).

### Exploratory factor analysis

Sample 2 was randomly divided in half into two groups, one for exploratory factor analysis and the other for validation factors. Exploratory factor analysis was performed on half of the randomly selected data (*n* = 416) using principal component analysis. The results showed that KMO = 0.874, χ^2^ value of Bartlett’s spherical test was 1,984.514, degree of freedom was 167, and significance probability value *P* < 0.01, indicating suitability for factor analysis. Principal component analysis was used to provide the common factors, and the principle of eigenvalues > 1 and the gravel plot showed that four factors were most suitable to be extracted. The eigenvalues of the four factors were 4.382, 2.263, 1.425, and 1.277, respectively, with a cumulative variance contribution of 57.812%. In terms of the loadings of the subfactors, the dimension of others’ emotion evaluation ranged from 0.671 to 0.826, the dimension of self-emotion management ranged from 0.739 to 0.903, the dimension of emotion use ranged from 0.653 to 0.910, and the dimension of social skills ranged from 0.726 to 0.835 (Table 2).

**TABLE 2:** Exploratory factor analysis of the Chinese version of SEIS (*n* = 416).

Dimensionality	Subject matter	Factor load	Eigenvalue (Math.)	Cumulative variance contribution
Emotional evaluation of others	SEIS1	0.767	4.382	19.563
	SEIS2	0.826		
	SEIS3	0.671		
	SEIS4	0.745		
Self-emotion management	SEIS5	0.881	2.263	36.612
	SEIS6	0.867		
	SEIS7	0.903		
	SEIS8	0.739		
Emotional use	SEIS9	0.875	1.425	42.958
	SEIS10	0.910		
	SEIS11	0.653		
social skills	SEIS12	0.802	1.277	57.812
	SEIS13	0.726		
	SEIS14	0.835		

### Confirmatory factor analysis

Based on the results of the exploratory factor analysis, Confirmatory factor analysis was performed on the other half of the administered sample data. And based on previous studies, we used some fit indices to assess the overall fit of the models; NFI, GFI, AFGI, CFI, and RMSEA. The values >0.90 for the CFI, NFI, GFI, and AFGI indicated an adequate fit; and the values <0.08 for the RMSEA indicated an adequate fit (Kline, 2011). The results of the analysis (Table 3) showed that χ^2^/df was less than 5, NFI, GFI, AFGI, and CFI were all greater than 0.9, and RMSEA was less than 0.08, and the model fit index met the fit criteria (Schmitt, 2011). As shown in Table 3, all standardized factor loading exceed 0.5, and the composite reliability (CR) of respective items was 0.897, 0.919, 0.865, and 0.892. The average variance extracted (AVE) of respective items was 0.687, 0.739, 0.681, and 0.734. According to Fornell and Larcker (1981), CR should exceed 0.6, and AVE should exceed 0.5 under ideal condition. Hence, all items for convergent validity were met.

**TABLE 3:** Confirmatory factor analysis of the Chinese version of SEIS (*n* = 416).

Dimensionality	Trails	Subject matter	Standardization factor	S.E.	AVE	C.R.
Emotional evaluation of others	→	SEIS1	0.853	-	0.687	0.897
		SEIS2	0.856	0.039		
		SEIS3	0.782	0.044		
		SEIS4	0.821	0.047		
Self-emotion management	→	SEIS5	0.824	–	0.739	0.919
		SEIS6	0.832	0.053		
		SEIS7	0.922	0.061		
		SEIS8	0.857	0.067		
Emotional use	→	SEIS9	0.872	–	0.681	0.865
		SEIS10	0.809	0.046		
		SEIS11	0.793	0.049		
Social skills	→	SEIS12	0.879	–	0.734	0.892
		SEIS13	0.826	0.082		
		SEIS14	0.865	0.088		

**Targets**	**χ ^2^/df**	**NFI**	**GFI**	**AFGI**	**CFI**	**RMSEA**
**Models**	3.74	0.917	0.945	0.913	0.926	0.062

**Dimensionality**			**1**	**2**	**3**	**4**
Emotional evaluation of others (1)			0.829			
Self-emotion management (2)			0.573	0.860		
Emotional use (3)			0.591	0.574	0.825	
Social skills (4)			0.492	0.641	0.583	0.857

The discriminant validity of the sports emotional intelligence (SEIS) was analyzed using the Fornell-Larcker criterion. That is, the analysis, i.e., whether the square root of the mean extracted variance of each dimension is greater than the correlation coefficient of that dimension with the other dimensions. The results show that the square root of the average extracted variance of each dimension is 0.829, 0.860, 0.825, and 0.857 respectively, which are all greater than the correlation coefficient between each dimension, indicating that the measurement model has good discriminant validity (Kline, 2011). Therefore, the Chinese version of the SEIS has good structural validity and meets the psychometric requirements.

### Criterion-related validity

With the development of positive psychology, researchers have made in-depth studies on the attribution of behavioral engagement and improvement strategies, among which, emotional intelligence has been proven to be an important factor influencing behavioral engagement. People with the highest levels of emotional intelligence have greater comprehension and capacity for regulation, have more skill in avoiding the influences of negative situations, show a tendency to maximize positive situations, and have more adaptive confrontation strategies along with a superior ability to resolve social problems (Gabriel et al., 2021). In sports contexts, people inevitably feel discomfort, frustration and helplessness (Zhao et al., 2016). Studies have shown that people with high sports emotional intelligence, who can correctly perceive and deal with the effects of negative emotions, will show higher activity, persistence and stability in exercise (Zhu et al., 2016). Studies found that the frequency of exercise correlated positively with sports emotional intelligence (Schutte et al., 2007), and sports emotional intelligence was positively related to exercise behavior (Eduardo et al., 2019). Therefore, we select the variables related to the exercise behavior as the Criterion-related variables.

The results of the correlation analysis between Chinese version SEIS and the Criterion-related validity are shown in Table 4. The results indicate that the total SEIS score, Emotional evaluation of others, self-emotion management, emotion use, and social skills were significantly and positively correlated with the total Exercise input score and its dimensions (vigorous persistence, focused satisfaction, value perception, and autonomy), total Exercise adherence score, total Exercise commitment score, and the dimensions (exercise commitment, exercise enjoyment, personal commitment, social constraint, and opportunity to participate) were significantly positively correlated (*p* < 0.05). This indicates that the Chinese version of the SEIS has high validity.

**TABLE 4:** Correlation coefficients of the Chinese version of SEIS and Criterion-related validity.

Calibration variables	Emotional evaluation of others	Self-emotion management	Emotional use	Social skills	Total SEIS score
Total exercise input score	0.563**	0.424*	0.560**	0.593**	0.551**
Vitality persistence	0.382*	0.346*	0.475**	0.461*	0.426*
Focus on satisfaction	0.317*	0.331*	0.374*	0.543**	0.467*
Perception of values	0.485**	0.447*	0.507**	0.472**	0.423*
Autonomy	0.450*	0.397*	0.436*	0.372*	0.355*
Total exercise adherence score	0.512**	0.459*	0.493**	0.418*	0.342*
Total exercise commitment score	0.478**	0.571**	0.325*	0.362*	0.452*
Exercise for fun	0.426*	0.545**	0.603**	0.428*	0.394*
Personal input	0.301*	0.420*	0.537**	0.485**	0.374*
Social constraints	0.482**	0.382*	0.375*	0.569**	0.573**
Participation opportunities	0.286*	0.255*	0.420*	0.347*	0.445*
Exercise commitment	0.378*	0.385*	0.412*	0.442*	0.393*

*P < 0.05, **P < 0.01.

### Reliability

The Cronbach’s alpha coefficient of the revised Chinese version of the SEIS was 0.873 and the Spearman-Brown split-half reliability was 0.822. The Cronbach’s alpha coefficient of the Emotional Appraisal of Others dimension was 0.849 and the Spearman-Brown split-half reliability was 0.827. The Cronbach’s alpha coefficient for the self-emotion management dimension was 0.905 and the Spearman-Brown half reliability was 0.891; the Cronbach’s alpha coefficient for the emotion use dimension was 0.858 and the Spearman-Brown half reliability was The Cronbach’s alpha coefficient for the social skills dimension was 0.824, and the Spearman-Brown split-half reliability was 0.807. The retest reliability of the SEIS was 0.890, with the others’ emotion evaluation dimension at 0.869, the self-emotion management dimension at 0.883, the emotion use dimension at 0.851, and the social skills dimension 0.816 (Table 5). In summary, the scale was tested to have a high reliability.

**TABLE 5:** Reliability analysis table of the Chinese version SEIS.

	Emotional evaluation of others	Self-emotion management	Emotional use	Social skills	Total SEIS score
Internal consistency reliability (*n* = 416)	0.849	0.905	0.858	0.824	0.873
Split-half reliability (*n* = 416)	0.827	0.891	0.836	0.807	0.822
Retest reliability (*n* = 83)	0.869	0.883	0.851	0.816	0.890

### Cross-gender equivalence test for the Chinese version of the SEIS scale

In order to examine the equivalence of the Chinese version of the SEIS across gender groups, multiple validation factor analysis was used to examine the morphological invariance, metric invariance and intercept invariance (Table 6; Roesch and Vaughn, 2006; Zhang and Bian, 2020). The results showed that the fit indices of morphological equivalence model, factor loading equivalence model and intercept equivalence model met the fit requirements. Meanwhile, the ΔCFI of the factor loading equivalence model and the morphological equivalence model were <0.01 and the ΔRMSEA was <0.01, and the ΔCFI of the intercept equivalence model and the factor load equivalence model were <0.01 and the ΔRMSEA was <0.01, indicating that the measurement equivalence of the Chinese version SEIE across gender groups was valid (Table 6).

**TABLE 6:** Cross-gender equivalence test in Chinese version of SEIS.

Models	χ ^2^	df	TLI	CFI	BIC	RMSEA (90% CI)
Morphological equivalence	432.61	142	0.922	0.934	0.064	0.054 (0.063, 0.081)
Factor load equivalence	448.59	152	0.921	0.933	0.064	0.054 (0.062, 0.081)
Intercept isometry	463.27	166	0.921	0.933	0.064	0.055 (0.062, 0.082)

Latent mean analysis (LMA) was also conducted on the sample two data to further analyze whether the differences in the means of the latent variables across gender groups were significant (Table 7). The results of the analysis showed that boys compared to girls, 0.085 higher on the others’ emotion evaluation dimension, 0.118 higher on the self-emotion management dimension, 0.072 higher on the emotion use dimension, and higher on the social skills dimension 0.136. The results of Cohen’s *d*-value showed that the others’ emotion evaluation dimension, the self-emotion management dimension, the emotion use dimension, and the social skills dimensions all have small degree of effect size. According to the criteria proposed by Xia et al. (2021), Cohen’s *d* value less than 0.2 is a low differential effect. Therefore, the results of the combined analyses indicated that there were no significant differences between the SEIE on the gender measures.

**TABLE 7:** Latent mean analysis.

Potential variables	Female student	Male student	Cohen’s *d*
Emotional evaluation of others	0.00	0.085	0.07
Self-emotion management	0.00	0.118	0.09
Emotional use	0.00	0.072	0.06
Social skills	0.00	0.136	0.11

## Discussion

Emotional intelligence harnesses and regulates and individual’s ability to perceive emotions in the surrounding environment, can overcome difficulties, promote a shift toward positive thinking and self-synchronization of goals (Lou and Jin, 2016), and also has a positive impact on behavioral engagement (Zheng et al., 2022). It has been shown that emotional intelligence implies the ability to develop self-motivation and promote a positive attitude toward life (Ubago et al., 2022), that differences in exercise engagement may be due to different levels of emotional intelligence in individuals (Zhu et al., 2016), and that for sport participants who experience unease, frustration or stress due to exercise goals or exercise execution beyond their situation, exercise emotional intelligence is a very important psychological resource that can help individuals regulate and positively control own emotions so that they positively influence sport behaviors and make effective decisions. At present, emotional intelligence in sport situations in China is mainly measured by educational psychology-related scales, such as the Emotional Intelligence Scale, Mayer-Salovey-Caruso Emotional Intelligence Test and Emotional Quotient Inventory. Although these classical instruments have high reliability and validity and are widely used in the field of parent discipline research, sports situations differ significantly from academic situations. There are some differences in the measurement content and structure of the two scales, thus leading to further bias in the measurement results. Therefore, there is a need for an instrument to measure emotional intelligence in sport situations (i.e., sports emotional intelligence). Based on this, the present study is based on this practical need and aims to revise the Chinese version of the Sports Emotional Intelligence Scale.

First, the scale of Nam (2015), which has the same structure for the concept of sports emotional intelligence, was translated according to the return-translation procedure. During the translation process, in order to minimize errors caused by cultural and environmental differences, the accuracy of the translated words was ensured in the translation-return-translation process, the content validity of the scale was improved, and the consistency of the semantic communication between the original and translated versions was confirmed in the completed final version. Second, in the item analysis, all entries had significant correlations with the mean scores of the subscales to which they belonged, which indicated that all entries had good discriminative power. In addition, after exploratory factor analysis, the revised Chinese version of the SEIS retained 14 items, including four dimensions of emotion evaluation of others (4 items), self-emotion management (4 items), emotion use (3 items), and social skills (3 items), which remained highly consistent with the structure of the original scale. The results of item analysis indicated that all 14 topics had high discriminatory power, and each topic was significantly and positively correlated with the total scale score, indicating that the items of the scale had high homogeneity with the scale as a whole. In foreign studies on sports emotional intelligence (Lane et al., 2009; Yoo, 2013; Choi and Kim, 2014; Nam, 2015), multidimensional factors of sports emotional intelligence have been investigated. Related studies provide support for this study.

In sports contexts, the emotional evaluation and understanding of others is not only directly related to one’s own emotions, but also to the understanding of the team’s emotions (Acebes et al., 2021b; Rubio et al., 2022). Evaluating the emotions of others is the ability to read and infer the mood of others and further emotional resonance. Emotional evaluation is a central concept in the cognitive theory of emotions. People evaluate each stimulus they encounter in terms of its relevance and meaning to themselves, and different evaluations lead to different emotions and thus different ways of coping (Kim, 2021). Research has shown that in team sports, the evaluation and understanding of one’s own and others’ emotions is very important (Kopp and Jekauc, 2019). The direct action in people’s initial evaluation is the outward expression of emotion, while the re-evaluation will be to determine the negative and positive meaning of the appearance of an emotional response and whether this emotional response is beneficial to physical exercise or sports competition (Sukys et al., 2019; Levillain et al., 2021). In sports contexts, people are stimulated by many factors and they evaluate stimuli according to their meaning and relevance to themselves. And the ability to evaluation and management the emotions of self, peers, and opponents is an important role in successful execution (Akelaitis and Malinauskas, 2018). In addition, it has been shown that emotion use can be expected to have a positive effect of sports emotional intelligence, i.e., to promote the maximization of positive emotions rather than the suppression of negative emotions (Lee et al., 2013). These results support the appropriateness of the structure of the subordinate factors of sports emotional intelligence embodied in the present study.

The results of the validity analysis on the Chinese version of the SEIS found that the total SEIS score, others’ emotion evaluation dimension, self-emotion management dimension, emotion use, and social skills dimension were significantly and positively correlated with exercise input, exercise adherence, and exercise commitment (*p* < 0.05). It shows that the revised Chinese version of SEIS has good Criterion-related validity. It also further validates the role of emotional intelligence with exercise in sport contexts. This is consistent with previous findings that sport emotional intelligence may increase sport performance and sport motivation (Sukys et al., 2019), exercise engagement (Ubago et al., 2019), and physical activity efficacy (Vaquero et al., 2020). Relevant research has demonstrated the positive effect of sports emotional intelligence on behavioral engagement (So, 2019). People with high emotional intelligence are often emotionally engaged in the task, which has a positive effect on task completion (Tinkler et al., 2021). In addition, in sport situations people with high emotional intelligence are better able to regulate their emotions, deal with real problems in a positive manner, and guide others to positive emotions (Zhao, 2021). This suggests a positive effect on exercise behavior by enhancing the sports emotional intelligence.

The multi-group validation factor analysis showed that the Chinese version of the SEIS had equal morphological equivalence, equal factor loadings, and equal intercepts for each entry for the gender latent variable, indicating that the scale has equal measurement significance across genders. The results of the latent mean analysis also revealed that the Chinese version of the SEIE scale differed in gender measures, but it was not significant. The differences found between levels of sports emotional intelligence is supported by previous studies (Laborde et al., 2017), and they encourage further study into the possible relationships between sports emotional intelligence and sports experience manifested themselves in the subgroups (Tak, 2007). The study showed that females are more emotionally sensitive to surrounding situations compared to males, and therefore have a slightly higher use of positive emotions as a method of enhancing executive performance (Fredrickson, 2004; Lee and Nam, 2012). Therefore, the result of slightly higher (non-significant) emotional intelligence ability in females than in males in this study is reasonable. Our results support the idea that different strategies according to gender should be considered in the context of sports to improve performance related sports emotional intelligence skills (Hanin, 2010; Campo et al., 2012).

The Chinese version of the SEIS has good reliability and validity, but in order to ensure the stability and general adaptability of the scale, it needs to be further validated by further expanding the test population (e.g., university athletes group, professional athletes group, primary and secondary school students group, etc.). Despite these limitations, our study has some implications for sports practice. It emphasizes the difference between measuring sports emotional intelligence and general emotional intelligence, an element that is generally forgotten when measuring this construct in China. Thus, in order to revise the SEIS that is universally applicable in China, provide a reliable measurement tool for conducting research on sports emotional intelligence measurement, and thus advance the development of sports emotional intelligence-related research in China.

## Conclusion

This study adds important information in measuring sports emotional intelligence, and provide evidence-based information for the measurement properties of Chinese SEIS. Findings from this study are consistent with those reported previously, and it can be concluded that the Chinese version of the SEIS has excellent test-retest reliability and acceptable construct and predictive validity in assessing sports emotional intelligence.

## Data availability statement

The raw data supporting the conclusions of this article will be made available by the authors, without undue reservation.

## Ethics statement

The studies involving human participants were reviewed and approved by the Ethics Committee of School of Education at Zhengzhou University. The patients/participants provided their written informed consent to participate in this study.

## Author contributions

JZ conceived, designed and performed the experiments, and wrote the manuscript. DB performed the experiments, wrote the manuscript, and prepared the tables. LQ reviewed the drafts of the manuscript, monitoring the whole process of the study. PS performed the experiments and prepared the tables. All authors contributed to the article and approved the submitted version.
