# Corrigendum: The development of the Chinese version of the Sports Emotional Intelligence Scale

**DOI:** 10.3389/fpsyg.2022.1068862

**Published:** 2022-10-27

**Authors:** Jia Zhang, Donghuan Bai, Long Qin, Pengwei Song

**Affiliations:** ^1^School of Physical Education, Chongqing University, Chongqing, China; ^2^School of Physical Education, Huaibei Normal University, Huaibei, China; ^3^School of Physical Education, Guangxi Science and Technology Normal University, Laibin, China; ^4^School of Physical Education, Keimyung University, Daegu, South Korea

**Keywords:** sports emotional intelligence, reliability, validity, scale revision, Chinese version

In the published article, there was a mistake in the “**Correspondence**” section. The Correspondence section was given as “Pengwei Song 740195450@qq.com” but should be “Jia Zhang zhangjiaaa@cqu.edu.cn; Pengwei Song 740195450@qq.com”.

The authors apologize for this error and state that this does not change the scientific conclusions of the article in any way. The original article has been updated.

## Publisher's note

All claims expressed in this article are solely those of the authors and do not necessarily represent those of their affiliated organizations, or those of the publisher, the editors and the reviewers. Any product that may be evaluated in this article, or claim that may be made by its manufacturer, is not guaranteed or endorsed by the publisher.

